# iRsp1095: A genome-scale reconstruction of the *Rhodobacter sphaeroides *metabolic network

**DOI:** 10.1186/1752-0509-5-116

**Published:** 2011-07-21

**Authors:** Saheed Imam, Safak Yilmaz, Ugur Sohmen, Alexander S Gorzalski, Jennifer L Reed, Daniel R Noguera, Timothy J Donohue

**Affiliations:** 1 Program in Cellular and Molecular Biology, University of Wisconsin - Madison, USA; 2 Department of Civil and Environmental Engineering, University of Wisconsin - Madison, USA; 3 Department of Bacteriology, University of Wisconsin - Madison, 5159 Microbial Sciences Building, 1550 Linden Drive, Madison, WI 53706, USA; 4 Chemical and Biological Engineering Department, University of Wisconsin - Madison, USA; 5 DOE Great Lakes Bioenergy Research Center, University of Wisconsin - Madison, USA; 6 BACTER Institute, University of Wisconsin - Madison, USA

## Abstract

**Background:**

*Rhodobacter sphaeroides *is one of the best studied purple non-sulfur photosynthetic bacteria and serves as an excellent model for the study of photosynthesis and the metabolic capabilities of this and related facultative organisms. The ability of *R. sphaeroides *to produce hydrogen (H_2_), polyhydroxybutyrate (PHB) or other hydrocarbons, as well as its ability to utilize atmospheric carbon dioxide (CO_2_) as a carbon source under defined conditions, make it an excellent candidate for use in a wide variety of biotechnological applications. A genome-level understanding of its metabolic capabilities should help realize this biotechnological potential.

**Results:**

Here we present a genome-scale metabolic network model for *R. sphaeroides *strain 2.4.1, designated iRsp1095, consisting of 1,095 genes, 796 metabolites and 1158 reactions, including *R. sphaeroides*-specific biomass reactions developed in this study. Constraint-based analysis showed that iRsp1095 agreed well with experimental observations when modeling growth under respiratory and phototrophic conditions. Genes essential for phototrophic growth were predicted by single gene deletion analysis. During pathway-level analyses of *R. sphaeroides *metabolism, an alternative route for CO_2 _assimilation was identified. Evaluation of photoheterotrophic H_2 _production using iRsp1095 indicated that maximal yield would be obtained from growing cells, with this predicted maximum ~50% higher than that observed experimentally from wild type cells. Competing pathways that might prevent the achievement of this theoretical maximum were identified to guide future genetic studies.

**Conclusions:**

iRsp1095 provides a robust framework for future metabolic engineering efforts to optimize the solar- and nutrient-powered production of biofuels and other valuable products by *R. sphaeroides *and closely related organisms.

## Background

Photosynthetic organisms perform many functions of significance to the planet and society. Plants and photosynthetic microbes are responsible for harvesting solar energy, evolving oxygen and sequestering atmospheric carbon dioxide [1]. In addition, algae, cyanobacteria and photosynthetic bacteria are either naturally able to or have been modified to evolve hydrogen (H_2_), accumulate oils and hydrocarbons, or produce alcohols or other compounds that can reduce society's dependence on fossil fuels [2,3]. The ability to understand, capitalize on or improve these activities is limited by our knowledge of the metabolic blueprint of photosynthetic organisms. To fill this knowledge gap, we are modeling the flow of carbon and reducing power in the well-studied photosynthetic bacterium *Rhodobacter sphaeroides*. This facultative bacterium is capable of either aerobic or anaerobic respiration, depending on the availability of oxygen (O_2_) or alternative electron acceptors. When O_2 _is absent or limiting, light energy can be harnessed by a photosynthetic electron transport chain that has features similar to those used by plants and other oxygen-evolving phototrophs [1]. During photosynthetic growth, *R. sphaeroides *is capable of autotrophic or heterotrophic growth using either carbon dioxide (CO_2_) or organic carbon sources [4,5]. Thus, it provides an ideal system for studying the details of each lifestyle and the mechanisms of transition between these various metabolic states.

*R. sphaeroides *has also received significant attention due to its biotechnological potential, with its ability to produce large amounts of carotenoids or isoprenoids as a source of biocommodities, H_2 _as a potential biofuel, or polyhydroxybutyrate (PHB) as raw material for biodegradable plastics [6]. Furthermore, the autotrophic metabolism of *R. sphaeroides *makes it a potential organism for use in the synthesis of chemicals or polymers that can serve as raw materials in the production of biofuels, or as a means of sequestering atmospheric or industrially-produced CO_2 _[2]. To understand and tap into the activities or products of this photosynthetic bacterium, detailed knowledge of its metabolic pathways is necessary. To provide this knowledge, we are generating computational models of the metabolic network of *R. sphaeroides *that are based on genomic information, which can be informed and integrated with laboratory analysis of wild type and mutant strains [3,7].

Over the last decade the field of constraint-based metabolic modeling has witnessed significant progress, which has led to major advances in the modeling, understanding and engineering of different biological systems [8-11]. As a consequence, high quality genome-scale metabolic reconstructions have been generated for many organisms [9]. These reconstructions serve both as structured databases of all the known and/or predicted metabolic functions of an organism and as the basis for the construction of mathematical models used in constraint-based analysis. The ability of constraint-based analyses to provide new biological insights has the potential to increase with the influx of high-throughput biological data sets [8,9]. Thus far, genome-scale reconstructions have been published for only one photosynthetic microbe, the oxygenic cyanobacterium *Synechocystis sp*. PCC 6803 [12-14]. Models of photosynthetic electron transport [15] and small scale *R. sphaeroides *metabolic networks that use flux balance analysis (FBA) [16] and ensemble modeling [17] have also been published.

Here we present iRsp1095, a manually curated genome-scale metabolic reconstruction for *R. sphaeroides *strain 2.4.1 consisting of 796 metabolites, 858 transformation reactions and 300 transport reactions. The reconstruction includes 1,095 genes, covering about 25% of the recognized *R. sphaeroides *open reading frames. To facilitate improved predictions, the biomass composition of *R. sphaeroides *was determined under a variety of growth conditions and used in generating biomass objective functions suitable for developing predictive models. FBA [18-20], flux variability analysis (FVA) [21] and alternate optima analysis [22,23] were used to predict metabolic fluxes under chemoheterotrophic (aerobic respiration), photoheterotropic and photoautotrophic (anaerobic) growth conditions. The predictive ability of iRsp1095 was validated by comparison with experimentally determined growth rate and fluxes of key metabolic products from continuous cultures. iRsp1095 was also used to predict metabolic flux distributions through key pathways including CO_2 _fixation and the electron transport chain. Overall, iRsp1095 shows good qualitative and quantitative agreement with experimental observations. Thus, iRsp1095 provides concepts and a basis for extensive future studies of this bacterium, other related bacteria and photosynthetic organisms in general.

## Results

### Model Reconstruction

The initial *R. sphaeroides *metabolic network was constructed by extracting genomic and metabolic information from KEGG [24], and combining this with results from metaSHARK [25] analysis (see Additional File 1 for details). We assigned directions to reactions in the network via a combination of thermodynamic and heuristic calculations/assumptions, which have been used previously [26] (see Additional File 1). The *R. sphaeroide*s model was further analyzed for stoichiometrically balanced cycles (SBCs) - internal network loops that carry flux in a closed system (i.e., when all exchange reactions are closed) with no net production or consumption of metabolites [20,27]. SBCs were manually eliminated from the network leading to the assignment of directionality to an additional 29 reactions in the network (see Additional File 1, Additional File 2 - Table S4). The remaining 150 (13%) reactions for which there was insufficient thermodynamic information were assigned as reversible. The directionality assignments in iRsp1095 are summarized in Table 1.

**Table 1:** Summary of the reaction directionality assignments in the model

	Number in each group	% of total Reactions
**Total Irreversible**	**401**	**35**
Thermodynamics only	109	9
Heuristics + Thermodynamics	125	11
ABC Transporter/tRNA charging	93	8
Spontaneous	8	1
Others*	66	6
**Total Reversible**	**757**	**65**
Thermodynamics/Heuristics	607	52
Unknown	150	13

**Total no. of reactions**	**1158**	

* Others includes groups of reactions assigned as irreversible based on SBC analysis or literature (e.g. other databases)

Gaps in the initial reconstruction, representing limitations in our current understanding of *R. sphaeroides *metabolism, were identified and filled (see Additional File 1). This process led to the addition of 30 transformation and 65 transport reactions to the network (see Additional File 2 - Table S11) and produced a model capable of predicting the production of biomass under defined conditions. FVA analysis with a completely open system (i.e., all exchange reactions allowed to carry flux) showed 140 blocked reactions remained at this stage, but these generally involved reactions (or pathways) required for the biosynthesis of low abundance end products (minor carotenoids and phospholipids) that are not considered as part of our biomass objective function. Thus, these 140 reactions are related to dead ends in iRsp1095.

### Formulation of biomass objective function

To obtain qualitative and quantitative outputs from constraint-based modeling using genome-scale models, the use of a meaningful objective function is critical [28]. Currently, the most widely used objective function in constraint-based modeling is the biomass objective function (BOF), as it represents a meaningful, though not necessarily accurate, ultimate goal of a microbial cell. While *R. sphaeroides *is a gram-negative bacterium, and in many respects similar to *E. coli *during aerobic growth, photosynthetic growth requires significant changes in metabolic machinery, and thus biomass composition, most notably in the pigment and lipid composition, as large amounts of chlorophyll or carotenoid pigments and phospholipids are contained in intracytoplasmic membrane (ICM) that houses the photosynthetic apparatus [29]. Thus, to generate representative BOFs for *R. sphaeroides*, we experimentally determined the major macromolecular constituents of aerobically and photosynthetically grown cells (Material and Methods). Based on these experimentally determined macromolecular components (Table 2), available genome sequence data [30] and published compositions of fatty acids and lipids [31-37], the BOFs were formulated as weighted combinations of precursors, with coefficients directly related to their percent composition of the biomass [20,38]. Details of the biomass calculations are contained in Additional File 3. The growth associated maintenance (GAM) energy requirement was estimated as previously described [20].

**Table 2:** Percent composition of cellular biomass of *R. sphaeroides *during photoheterotrophic and aerobic growth*

Components	% Composition of biomass (Photo)	% Composition of biomass (Aero)
DNA	1.9	2.8
RNA	5.1	7.1
Protein	53.6	49.3
Lipids^a^	17.1	12.8
PHB	10.4	17.6
Bacteriochlorophyll	0.4	0
Carotenoids	0.1	0
Glycogen	1.4	0.4
Lipopolysaccharides^b^	3	3
Cell Wall^b^	2	2

* Inorganic fraction estimated to be about 5% of biomass [66].

^a ^Lipid composition of *R. sphaeroides *consisting of phospholipids and sulfolipids.

^b ^Estimated from *E. coli *cell wall and lipopolysaccharides percent contribution.

### Overview of iRsp1095

**Figure 1:**
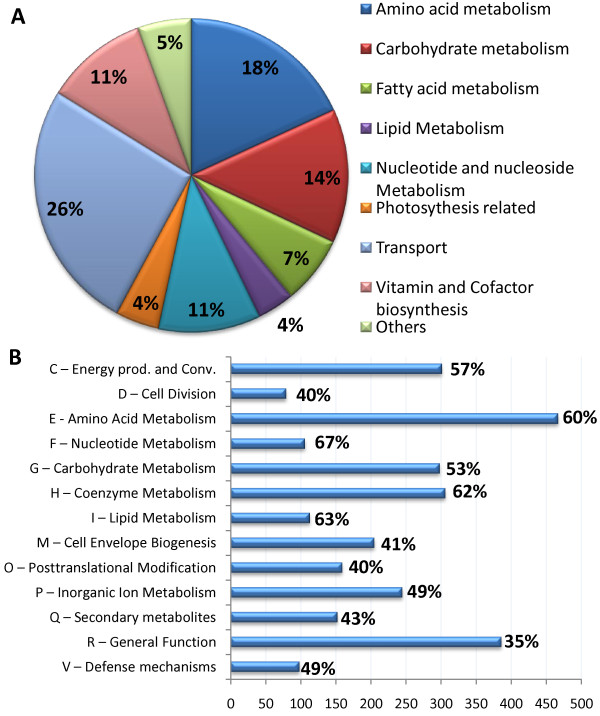
**Distribution of reactions and gene products in iRsp1095**. (A) The pie chart depicts the subsystem distribution of the reactions in iRsp1095, with the percent contribution of each subsystem of reactions indicated in the corresponding section of the chart. It can be seen that amino acid, carbohydrate and nucleotide metabolism dominate the enzymatic reactions present in iRsp1095, while photosynthesis related reactions represent a smaller but significant fraction. (B) The bar chart depicts the distribution of gene products in iRsp1095 based on COG classification with the percent coverage shown for each class. Only COG classes significantly enriched for proteins present in the model (p < 0.01, hypergeometric distribution) are shown.

iRsp1095 consists of 796 unique metabolites, 858 transformation reactions, 300 transport reactions and 148 exchange reactions (Table 3). The list of reactions, metabolites, thermodynamic calculations, genes and references used are in Additional File 2. The network is divided into 3 compartments (extracellular, periplasmic and cytoplasmic), with appropriate transport reactions across the outer and inner membranes. Individual metabolites, including cytoplasmic, periplasmic or extracellular instances of a given metabolite, were given reconstruction-specific unique identifiers for internal use, which were mapped to other database identifiers (PubChem, Cas, KEGG and BiGG). The iRsp1095 reconstruction accounts for 1,095 genes representing ~25% of the annotated *R. sphaeroides *open reading frames. Of the 1158 reactions in iRsp1095, 1,049 (90.6%) have gene-protein-reaction (GPR) assignments, with 203 of these having associated experimental data, while 95 (8.2%) of the reactions without GPR assignments correspond to place holder reactions for which a putative gene could not be assigned. The remaining 14 reactions correspond to known spontaneous or diffusion reactions (Table 3, see Additional File 2 - Table S1). The breakdown of the sub-system distribution of the reactions is shown in Figure 1a. Analysis of the distribution of the gene products in iRsp1095 using cluster of orthologous groups (COGs) classification [39], shows that 13 of the 22 COG categories are significantly enriched for the proteins present in the model (p-value < 0.01, hypergeometric test), with amino acid metabolism having the highest number and nucleotide metabolism showing the greatest coverage (Figure 1b). The genome-scale reconstruction was converted into a stoichiometric matrix consisting of 796 rows and 1309 columns, including exchange reactions to allow metabolites to be taken up or secreted in to the extracellular space, as well as 3 demand reactions for key metabolites not included in the biomass reaction (PHB, glycogen and minor carotenoids) (Table 3). The equivalent SBML format of the model was generated for distribution and potential use in other modeling environments (see Additional File 4). This file has been deposited in the BioModels database [40] (accession: MODEL1106220000).

**Table 3:** Overview of iRsp1095

Categories	No. in the reconstruction	
**Genes**	**1095**	
ORFs	1049	96.0%
tRNA genes	46	4.0%

**Metabolites**	**1096**	
Unique metabolites	796	
Cytoplasmic	795	
Periplasmic	151	
Extracellular	150	

**Reactions**	**1158**	
Enzymatic Reactions	858	74.1%
Transport reactions	300	25.9%
Reactions Associated with genes	1049	90.6%
Reactions based on experimental evidence	203	17.5%
Reactions inferred based on gene homology	846	73.1%
Spontaneous/Diffusion reactions	14	1.2%
Reactions without gene association	95	8.2%
Reactions associated with multi-protein complexes	130	11.2%
Reactions associated with isozymes	262	22.6%
Reversible Reactions	757	65.4%
Irreversible Reactions	401	34.6%
Exchange Reactions	148	
Demand Reactions	3	

### Model validation

We used FBA and other constraint-based approaches to interrogate the properties of the iRsp1095, with simulations conducted for aerobic respiration, dark anaerobic respiration in the presence of the electron acceptor dimethyl sulfoxide (DMSO) and photoheterotrophic growth (anaerobic growth in the presence of light and an electron-rich carbon source) using Sistrom's minimal media (SIS) [41] containing one of a variety of carbon sources (see Additional File 2 - Table S9). Photoautotrophic growth with CO_2 _as the sole source of carbon and H_2 _or hydrogen sulfide (H_2_S) as the electron donor was also simulated.

#### Qualitative Assessment of Metabolic model

As a first step in assessing the performance and breadth of iRsp1095, we used FBA to test for the ability of the model to predict the production of biomass and H_2 _while supplied with SIS minimal media. The model was capable of predicting growth in the dark in the presence of O_2 _or DMSO as known electron acceptors, under photoautotrophic conditions using CO_2 _as the sole carbon source and either H_2 _or H_2_S as electron donor, and photoheterotropically with a variety of organic carbon sources (Table 4). In addition, when the ability to utilize the various carbon, nitrogen, phosphorus and sulfur sources present in iRsp1095 was tested, it predicted photosynthetic growth on 129 potential carbon sources, 72 potential nitrogen sources, 46 potential phosphorus sources and 9 potential sulfur sources. While no high throughput phenotypic screens have been conducted for *R. sphaeroides*, growth on 25 of the carbon sources predicted by iRsp1095 to support net biomass formation (~20%) have previously been reported [6,42,43] (see Additional File 2 - Table S6), while those carbon sources not yet tested as growth substrates in the literature provide candidates for future validation and correction of the model.

**Table 4:** Growth phenotypes predicted by the model under a variety of routinely utilized laboratory conditions*

	Light	Dark		
		**Electron Acceptor**		
		
		**O**_**2**_^**a**^	**DMSO**^**a**^	**None**

Succinate + NH_3_	+/+^b^	+/-	+/-	-/-
Succinate + Glutamate	+/+ ^b^	+/-	+/-	-/-
Lactate + NH_3_	+/+ ^b^	+/-	+/-	-/-
Glutamate only	+/-	+/-	+/-	-/-
CO_2 _+ H_2 _+ NH_3_	+/-	-/-	-/-	-/-
CO_2 _+ H_2 _+ N_2_	+/-	-/-	-/-	-/-

* +/+ Growth and H_2 _production predicted; +/- Growth but no H_2 _production predicted; -/- No growth

^**a **^Oxygen (O_2_) or DMSO was used as the sole electron acceptors in simulations.

^b ^Succinate and lactate uptake rates were set to 3 mmol/g DW h, while the NH_3 _and glutamate uptake rates were set to 1 mmol/g DW h, as these are within the rate of experimentally observed uptake rates for these substrates. CO_2_, H_2 _and N_2 _uptake rates were set to 1 mmol/g DW h for photoautotrophic growth simulations.

**Figure 2:**
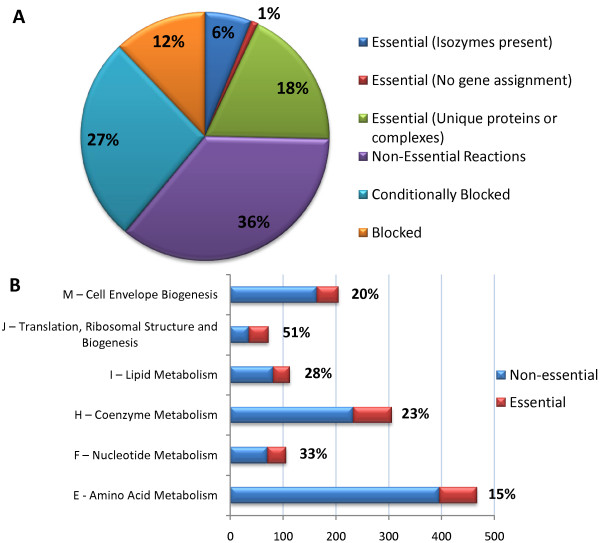
**Summary of gene and reaction essentiality analysis**. (A) Categorization of the reactions present in the model based on their requirement for growth in SIS minimal media supplemented with succinate. About 25% of the reactions in the model are known or predicted to be required for growth under these conditions. (B) Distribution of essential gene products by COG functional classes. The total number of gene products in each class (essential + non-essential) represent only those present in the current model. The percentage of essential proteins in each class is indicated. COG classes depicted are those significantly enriched for essential gene products (p < 0.01, hypergeometric distribution).

An extensive set of *R. sphaeroides *mutants does not currently exist for validation of gene knock-out simulations using iRsp1095. However, gene essentiality analysis still allows us to generate hypotheses about genes and reactions that are potentially essential under one or more growth conditions. We used FBA to conduct single reaction and gene deletion analyses during simulations of photoheterotrophic growth using succinate as a carbon source and ammonia as the nitrogen source (with light uptake left unconstrained). Under these conditions, iRsp1095 predicts that a core set of 293 reactions (25% of the network) are essential for growth (Figure 2a). Seventy of these "essential" reactions are associated with isozymes and thus would potentially require multiple gene deletions to inactive the cognate pathway. FVA analysis at optimal growth rate, predicts that 415 (36%) of the reactions in the network are capable of carrying flux, but are not essential for growth on minimal media containing succinate as a carbon source. An additional 310 (27%) of the reactions are predicted to be incapable of carrying flux during photoheterotrophic growth on succinate and ammonia and correspond to transport and transformation steps not required under these conditions but could potentially be essential under alternative growth conditions. The remaining 140 (12%) of the reactions in the network cannot carry flux under any of the conditions tested (i.e. blocked reactions). Furthermore, single gene deletion analysis showed that 217 (20%) of the 1095 genes present in iRsp1095 were essential for growth under these conditions (see Additional File 2 - Table S10). The distribution of these gene products based on COG classification is shown in Figure 2b.

#### Quantitative Assessment of iRsp1095

**Figure 3:**
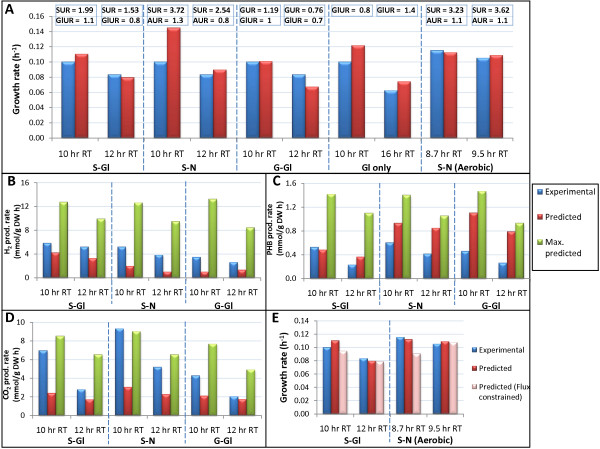
**Quantitative assessments**. The bar graphs compare experimentally measured (a) growth rate, (b) H_2 _production, (c) PHB production and (d) CO_2 _production with predictions made by iRsp1095. Growth conditions included photoheterotrophic growth on the following carbon and nitrogen sources: succinate + glutamate (S-Gl), succinate + NH_3 _(S-N), glucose + glutamate (G-Gl), glutamate as a sole source of carbon and nitrogen (Gl only) and aerobic growth on succinate + ammonia (S-N (Aerobic)). The experimentally determined succinate uptake rate (SUR), ammonia uptake rate (AUR), glucose uptake rate (GUR) and glutamate uptake rate (GlUR) (all in mmol/g DW h) used in all simulations are provided in the boxes above the bars in Figure 3a. The FVA predicted maximum production rate of each of the metabolites during optimal growth (Max. predicted) is also provided. In each case the FVA predicted minimum production rate was zero. It should be noted that while ammonia can inhibit nitrogenase activity and thus H_2 _production, the ammonia uptake rates in S-N chemostats are not high enough to inhibit nitrogenase activity, hence the production of H_2 _under these conditions. No detectable amounts of PHB, H_2 _or CO_2 _were produced during growth on glutamate only, which is in line with model predictions. Figure 3e shows the decreases in predicted growth rate for S-Gl and S-N (Aerobic) conditions upon constraining the model with experimentally observed fluxes for H_2_, PHB and/or CO_2 _during simulations (Predicted (flux constrained)). The predicted optimal growth rates for other conditions shown in Figure 3a were unaffected by imposing these fluxes.

To assess iRsp1095 quantitatively, we used FBA and alternate optima analysis to sample the feasible solution space and make predictions about specific growth rate and the rate of production of key metabolic products during photoheterotrophic growth on a variety of carbon and nitrogen sources (succinate + ammonia, succinate + glutamate, glucose + glutamate and glutamate only), as well as during aerobic growth on succinate and ammonia. We compared the predicted fluxes to experimentally determined growth rate and production rates for these key metabolites during *R. sphaeroides *growth in continuous culture. The model was constrained with experimentally determined uptake rates for the various carbon and nitrogen sources, while being freely allowed to take up all other media components, as well as absorb light. We found that iRsp1095 was capable of accurately predicting cellular growth rate, with predictions generally within 0.4 - 25% of the experimentally observed growth rate (Figure 3a), with an overall correlation of 0.75 (P = 0.012) across the conditions tested. The FBA predicted growth rate is generally slightly higher than that observed experimentally (especially during growth on succinate + NH_3_). These observed differences could be the result of several factors, including stress and feedback inhibition, which cannot be captured in stoichiometric models. Furthermore, many laboratory strains are not necessarily evolved for maximization of growth and thus do not meet the FBA predicted growth rate prior to adaptive evolution experiments [44]. Nevertheless, the predicted growth rates are closer to experimental observations than results previously seen in some other organisms [45,46], suggesting *R. sphaeroides *strain 2.4.1 is not as far from optimal growth under the conditions we analyzed.

Solutions to linear programming problems are not always unique [21], thus several distinct flux distributions could potentially result in the predicted optimal growth rate. To search the feasible solution space for the possible optimal solutions achievable by iRsp1095 given the constraints on substrate uptake rates, we used a mixed integer linear programming (MILP)-based alternate optima algorithm [22,23]. A small subset of the reactions in iRsp1095 predicted to function as sinks for excess reducing power were used in sampling the optimal subspace (Materials and Methods). This analysis led to the identification of some 2 - 17 equivalent optimal solutions, across the various conditions tested, that differed in their pattern of flux distributions. The optimal solution with fluxes for H_2_, PHB and CO_2 _presented in Figure 3b, 3c and 3d respectively, represents one where non-zero fluxes for all 3 metabolites were observed in the same solution and which most closely matched the observed experimental data. In addition, the FVA predicted maximum and minimum production rates of these metabolites were assessed. Overall, the predicted amounts of H_2_, PHB and CO_2 _generally ranged from within 4% to 200% of the experimentally measured fluxes (Figure 3b,c and 3d). Furthermore, constraining the model with the experimentally observed fluxes for PHB, H_2 _and CO_2 _did not result in decreases in the maximum predicted growth rate for most cultures, suggesting that these experimentally determined flux distributions are also within the optimal subspace. However, applying these constraints to simulations of photoheterotrophic growth on succinate + glutamate and aerobic growth on succinate + NH_3 _decreases the predicted growth rate, suggesting the organism is growing sub-optimally under these conditions (Figure 3e). Overall, the experimentally measured fluxes generally fell within the optimal solution space of our simulations.

#### Sensitivity analysis

Further analyses were conducted to evaluate the effects of BOF composition, light uptake and P/O ratio on growth and metabolite production rates in iRsp1095 (see Additional File 5). These analyses showed that: (i) growth rate predictions are not significantly affected by changes in BOF composition, however the production rate of certain metabolites (e.g., H_2_) can be affected (see Additional File 5 - Figure S1); (ii) the predicted growth rate and production rates for PHB and H_2 _increased with increasing light until they reached a plateau, while the predicted CO_2 _production decreased with light uptake, presumably reflecting improved carbon assimilation as biomass increased (see Additional File 5 - Figure S2); and (iii) the P/O ratio can have a significant impact on growth rate, as seen in other metabolic models [38] (see Additional File 5 - Figure S3).

### Evaluation of H_2 _Production by *R. sphaeroides*

H_2 _serves as a major electron sink for the dissipation of excess substrate reducing power during anoxic phototrophic growth in *R. sphaeroides *[3]. H_2 _production in *R. sphaeroides *mainly results from nitrogenase activity, through the coupling of N_2 _fixation with H_2 _production [47]. However, nitrogenase will also reduce protons, producing H_2 _when N_2 _is absent [48]. Since high levels of ammonium inhibit nitrogenase activity, H_2 _production can be stimulated by supplying the culture with an alternative nitrogen source, such as glutamate [49]. While there is no evidence of H_2 _production by the hydrogenase of *R. sphaeroides*, H_2 _accumulation in *R. sphaeroides *cultures can also be affected by the presence of this enzyme if H_2 _is reutilized by the cells [47].

**Figure 4:**
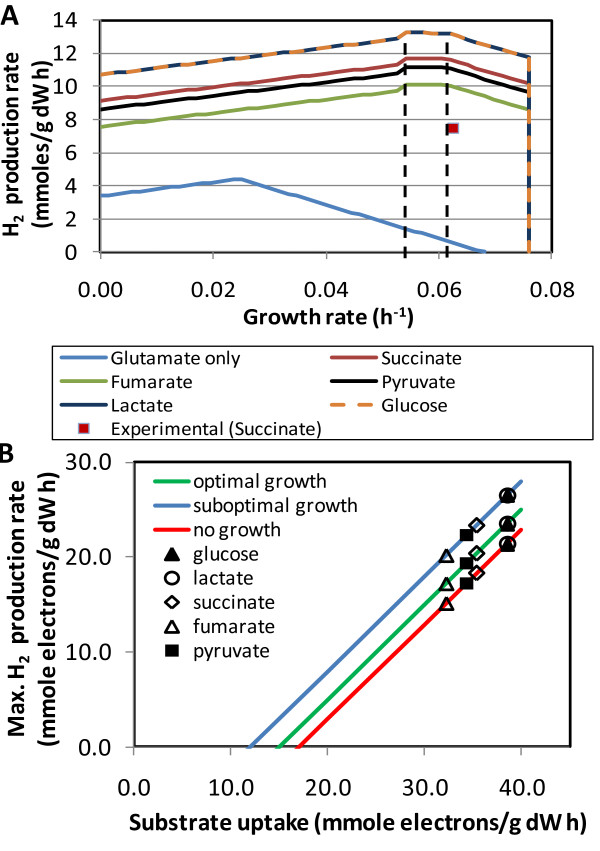
**H**_**2 **_**production potential of iRsp1095 with different carbon sources and glutamate as the nitrogen source**. (a)Relationship between growth rate and H_2 _production. Uptake rates were determined based on a reference chemostat (16 h retention time) fed with succinate (SUR = 1.57 mmol/g DW h) and glutamate (GlUR = 0.75 mmol/g DW h), such that, GlUR was not varied and any other carbon source was supplied by keeping the total number of carbon atoms constant. Experimentally observed H_2 _flux from the reference chemostat is shown as a data point. Dashed lines indicate the region that represents the suboptimal growth rates at which the theoretical maximum amount of H_2 _is produced for each carbon source. (b)Relationship between substrate reducing power and electrons used in H_2 _production (glutamate only case not included). Best fitting lines correspond to three phases of growth in (a) (R^2 ^= 1 in all cases).

As a specific application of iRsp1095 we evaluated H_2 _production when *R. sphaeroides *is grown on one of several carbon sources with glutamate used as the only nitrogen source, under anoxic photosynthetic conditions. Figure 4a shows sensitivity plots of the relationship between growth rate and H_2 _production capacity. The theoretical maximum H_2 _production while maximizing growth is achieved at the optimal growth rate of 0.076 h^-1 ^(e.g., 10.2 mmol/g DW h for succinate). However, for all carbon sources tested the theoretical H_2 _production maxima were reached under suboptimal growth conditions with biomass fluxes around 0.055-0.060 h^-1^. For comparison to experimental production rates, a reference chemostat yielded a H_2 _flux that was about two thirds of the theoretical maximum of 11.5 mmol/g DW h (Figure 4a). For cells using glutamate as the sole source of carbon and nitrogen (Figure 4a), iRsp1095 predicts little to no H_2 _production near maximum growth, consistent with our experimental observations with glutamate only cultures, which produced no detectable H_2_.

In Figure 4a, the maximal value derived by iRsp1095 was predicted to be larger for more reduced compounds (lactate and glucose) and smaller for less reduced carbon sources (fumarate and pyruvate). Thus, an important question is whether maximum H_2 _production is a function of the substrate reducing power only or is also affected by substrate-specific pathways. To address this question, we converted substrate uptake and H_2 _production rates to electron fluxes using the stoichiometry of half reactions for electron donation and acceptance [50]. We found that the maximum H_2 _production potential for growing cells was linearly related to the available electrons from the substrates (carbon source and glutamate) as shown in Figure 4b (see below for the no-growth condition). The linear trend indicates that H_2 _producing capacity is proportional to substrate reducing power, irrespective of the carbon source. The intercept of this relationship, where no electrons are available to support H_2 _production, shows the reducing power that supports growth alone. The derived slope, which equals 1, indicates that maximizing H_2 _production can theoretically be achieved by directing all electrons in excess of that required for growth to H_2 _production. This is a significant finding since there are multiple competing pathways that can dissipate substrate reducing power, so this result suggests that H_2 _production can be increased from experimental values to theoretical maxima if these other pathways are silenced.

An interesting prediction from these data (Figure 4) is that growing cells can support a larger H_2 _production potential than resting cells, since, in all cases studied, metabolism with no flux in the biomass reaction yielded the lowest maxima of H_2 _flux. Therefore, the breakdown of substrates in biomass synthesis pathways seems necessary to provide maximal reducing power for H_2 _production. The relationship of theoretical maxima at the no-growth condition to the reducing power of the substrates was similar to those with growing cells (Figure 4b). That is, the slope of the no-growth curve was also equal to 1, indicating that H_2 _can be theoretically maximized when all excess electrons are converted to H_2_. However, the model also predicted a baseline of reducing power not converted to H_2_, which is represented in Figure 4b by the intercept of the no-growth line with the horizontal axis. The flux distribution output from iRsp1095 suggests H_2_S as the product accumulating this reducing power baseline.

### Metabolic flux distributions

We used FBA to predict metabolic flux distributions during aerobic, photoheterotrophic and photoautotrophic growth.

#### Photoautotrophic growth

As expected, during photoautotrophic growth iRsp1095 predicts there is a high flux through ribulose-1,5-bisphosphate carboxylase/oxygenase (RubisCO) and the Calvin cycle, as it represents a major pathway for CO_2 _assimilation [17,51]. However, previous analysis of *R. sphaeroides *has shown that a RubisCO mutant (in which form I and form II RubisCO have been deleted) is still capable of photoautotrophic growth, when using less reduced electron donors than H_2 _(e.g., thiosulfate or sulfide) [51], suggesting that alternative CO_2 _assimilation pathways can support growth under these conditions. Pyruvate carboxylase has previously been shown not be one of these alternative routes [7]. iRsp1095 predicts that the ethylmalonyl pathway, involved in acetyl-coA assimilation, is a candidate for CO_2 _sequestration under these conditions. The first enzyme in this pathway, crotonyl-CoA carboxylase/reductase, catalyzes the reductive carboxylation of crotonyl-CoA to ethylmalonyl-CoA [52,53] and iRsp1095 predicts this pathway can carry sufficient flux for photoautotrophic growth in the absence of RubisCO. Only when the flux through both the RubisCO and crotonyl-CoA carboxylase/reductase reactions are set to zero in the model, does photoautotrophic growth with thiosulfate or sulfide cease to be predicted by iRsp1095, suggesting it is potentially an alternative route of CO_2 _fixation in *R. sphaeroides*, and the only one currently incorporated in the model that is capable of supporting photoautotrophic growth in the absence of RubisCO.

#### Photoheterotrophic growth

**Figure 5:**
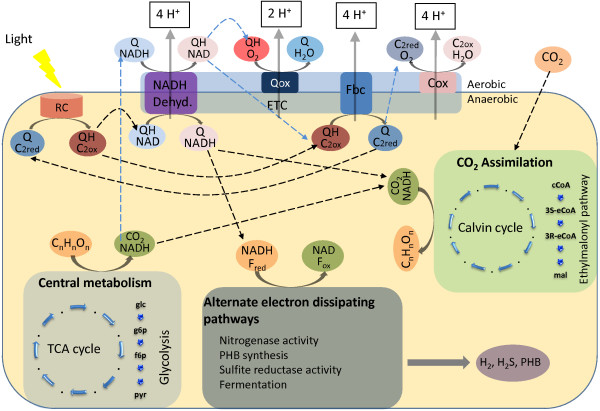
**Overview of the flux distributions under various growth conditions**. Figure shows the flow of electrons during aerobic (light blue dashed arrows) and photosynthetic (black dashed arrows) growth. During anaerobic growth electrons flow from the photosynthetic reaction center (RC), along the electron transport chain (ETC) and back to the RC via reduced cytcochrome (C2_red_) in a cyclic photosynthetic system [54], with protons pumped into the periplasm. NADH dehydrogenase is predicted to function in the reverse direction, reducing NAD to NADH while oxidizing ubiquinol (QH) to ubiquinone (Q). Under these conditions, excess electrons obtained from the oxidation of electron rich carbon sources (C_n_H_m_O_p_) would be dissipated via the alternative electron consuming pathways or via carbon fixation. During aerobic growth either cytochrome oxidase (Cox) or quinol oxidase (Qox) can be used in the oxidation of QH to Q, with the Cox reaction favored as more protons are pumped across the cytoplasmic membrane. Under these conditions, NADH dehydrogenase functions in the forward direction oxidizing NADH to NAD. ATP synthase is omitted from the ETC for simplicity. C_2ox _- oxidized cytochrome; F_red _- reduced ferredoxin; F_ox _- oxidized ferredoxin.

FBA simulation of photoheterotrophic growth on succinate and ammonia predicts metabolic flux through reactions involved in the TCA cycle, as might be expected, with significant amounts of H_2 _being produced as the rate of ammonia uptake used in simulation (1 mmol/g DW h) results in nitrogen limiting conditions, allowing excess succinate supplied to the model to be converted to H_2_. iRsp1095 does not predict flux through RubisCO to be essential for photoheterotrophic growth; however, it is known that RubisCO is essential for photoheterotrophic growth of wild-type *R. sphaeroides *on carbon sources like succinate and malate, where there is reductive assimilation of CO_2 _[7]. Alternate optima analysis [22,23] predicts a few FBA optima exist wherein RubisCO is used as a major electron sink, however other FBA optima predict the extensive utilization of one or more alternative pathways to recycle excess reducing power including: (i) nitrogenase activity resulting in the production of large amounts of H_2_; (ii) the sulfite reductase reaction resulting in the production of H_2_S; (iii) PHB synthesis; or (iv) the use of the ethylmalonyl pathway (Figure 5). Previous, analyses of *R. sphaeroides *RubisCO mutants have shown that cells are capable of reprogramming their regulatory network to restore photoheterotrophic growth on electron-rich carbon sources [7]. The alternative reactions known to be utilized to restore photoheterotrophic growth under these conditions include nitrogenase reaction yielding H_2 _and sulfate reduction to H_2_S [7,17]. Thus the observed alternate optima predicted in iRsp1095 likely represent distinct functional states, all achievable by *R. sphaeroides *based on its metabolic capabilities, but the wild type organism is largely restricted to only a limited number of these as a result of its complex and highly evolved regulatory network, which keeps most of these other functional states silent in the absence of perturbation. Given that these regulatory constraints are not present in iRsp1095, the majority of these functional states are thus achievable, allowing for the prediction of growth in the absence of RubisCO.

Analysis of electron transport chain activity during photoheterotrophic growth shows significant flux through ubiquinol-cytochrome c reductase (Fbc complex) and NADH dehydrogenase, with both enzymes predicted as being essential during growth on succinate and ammonia. The essentiality of the Fbc complex might be expected as it serves as the only means of providing reduced cytochromes required for the photosynthetic light reaction [1,54]. In contrast, the requirement for NADH dehydrogenase activity during photoheterotrophic growth on succinate is proposed to reflect the need to oxidize ubiquinol and generate NADH for anabolic reactions [55]. Indeed, during anaerobic growth, iRsp1095 predicts that NADH dehydrogenase uses the transmembrane electron potential to drive the oxidation of ubiquinol to ubiquinone and the concomitant reduction of NAD+ to NADH (Figure 5), thus freeing up ubiquinone for use in the cell, while providing NADH for biosynthetic reactions. Furthermore, iRsp1095 predicts that addition of DMSO would restore photoheterotrophic growth in the absence NADH dehydrogenase, as might be expected if cells lacking this enzyme were unable to balance electron flux. It should be noted however, that the predicted essentiality of NADH dehydrogenase during photoheterotrophic growth appears to be conditional, as iRsp1095 predicts that growth occurs with other carbon sources which apparently have less of a requirement for NADH dehydrogenase activity.

#### Aerobic growth

FBA simulations of aerobic respiratory growth on succinate and ammonia predict significant flux through the TCA cycle and reactions specific to succinate metabolism with the concomitant production of large amounts of CO_2 _and trace amounts of urea. iRsp1095 also predicts that cyctochrome c oxidase (Cox) activity is sufficient and required for optimal aerobic respiratory growth. In the absence of Cox activity, quinol oxidase (Qox), which is capable of ubiquinol oxidation to ubiquinone coupled to direct O_2 _reduction, is predicted to support aerobic respiratory growth, but the predicted growth rate in this mutant is only 60% of the predicted optimum. A similar reduced growth rate is also predicted in the absence of the Fbc complex, as this also results in flux being directed through Qox in order to oxidize ubiquinol (Figure 5). This observed reduction in growth rate might be expected as flux through the Fbc and the Cox complexes pumps 8 protons across the membrane, while flux through Qox, which bypasses both enzymes, results in only 2 protons being pumped across the membrane, thus providing much less energy for the cell (Figure 5). Interestingly, NADH dehydrogenase, which is predicted by iRsp1095 to be essential during photoheterotrophic growth on succinate and ammonia, is not predicted to be essential during aerobic respiration. Indeed, only an ~8% decrease in growth rate is predicted during aerobic respiration in the absence of NADH dehydrogenase activity. Since ubiquinol can be oxidized either via the Fbc-Cox pathway or Qox, NADH dehydrogenase activity is no longer required these conditions. Thus, iRsp1095 predicts that NADH dehydrogenase functions in NADH oxidation during aerobic respiration and contributes to formation of a proton gradient across the membrane (Figure 5).

## Discussion

Previous research has shown the potential of constraint-based analysis for understanding metabolic networks [9]. Given the well-studied photosynthetic lifestyle and biotechnological potential of *R. sphaeroides*, iRsp1095 provides an enabling framework that should increase our understanding of and ability to improve its metabolic machinery. One of the major challenges faced by photosynthetic and many other bacteria is the need to balance the generation of reducing equivalents obtained from light or carbon sources with pathways that consume these electrons. Previous analysis has shown that *R. sphaeroides *partitions significant proportions of reducing equivalents into cellular biomass, PHB, excreted organic acids or H_2 _[3]. Furthermore, genetic analysis suggests that CO_2 _fixation via RubisCO is also essential for recycling excess reductant during photoheterotrophic growth. Analysis of the flow of reducing equivalents in iRsp1095 reveals that *R. sphaeroides *has several alternate means to potentially recycle reducing equivalents, but not all of these are functional in wild type cells. In addition to known processes like CO_2 _fixation via RubisCO, PHB synthesis and H_2 _production [3,7,17], iRsp1095 also predicts H_2_S production by sulfite reductase activity, reductive carbon assimilation via the ethylmalonyl pathway and secretion of metabolites (e.g., lactate and formate) as alternative routes for dissipating excess reducing power. While the role of some of these processes have been experimentally verified [17], others represent novel predictions. The dependence of wild type *R. sphaeroides *on RubisCO for photoheterotrophic growth, a phenotype not corroborated by iRsp1095, suggests that these alternative routes for dissipating excess reducing power could either represent silent functional states or are insufficiently active to support growth in the absence of the Calvin cycle.

Our evaluation of H_2 _production potential of *R. sphaeroides *with iRsp1095 showed that continuous culture performance reached two-thirds of the predicted maximum H_2 _production (Figure 4). To harness the remaining potential predicted by iRsp1095, pathways that contribute to and compete with H_2 _production needed to be determined. Interestingly, biomass synthesis is predicted by iRsp1095 to be a contributor to H_2 _production. Therefore, we analyzed the pathways that divert electrons from H_2 _production in growing cells, when succinate and glutamate were the substrates. This analysis predicts a set of reactions (Table 5) whose collective elimination would yield a H_2 _production rate of 11.3 mmol/g DW h, very close to the theoretical maximum of 11.5 mmol/g DW h that was predicted by iRsp1095. Five of the products in Table 5 are intermediates in cell synthesis pathways and cannot compete with H_2 _production under optimal growth conditions (i.e., when biomass flux is maximized). Hence, these reactions provide predictions on the pool of electrons that can be diverted from biomass synthesis to H_2 _production.

**Table 5:** Key electron sinks that compete with H_2 _production**

Electron Sink	Pathway	Reaction	Responsible Genes
PHB	Butanoate Metabolism	RXN0589	RSP0382, RSP1257
H_2_S	Sulfur Metabolism	RXN0866	RSP1942
Glycogen	Starch & Sucrose Metabolism	RXN0849	RSP2887
Formate	One Carbon Pool by Folate	RXN0323	RSP0944
Glycerate	Glycerolipid Metabolism	RXN0030	RSP1292, RSP1507, RSP3740, RSP4003, RSP2372
4-Coumarate	Nitrogen Metabolism	RXN0495	RSP3574
Methanethiol	Cysteine & Methionine Metabolism	RXN1096	RSP1851
4-Aminobutyraldehyde*	Glycolysis/Gluconeogenesis	RXN0031	RSP4003, RSP3740, RSP2372, RSP1507, RSP1292
Chitobiose*	Amino Sugar and Nucleotide Sugar Metabolism	RXN0040	RSP2941
D-1-Aminopropan-2-ol O-phosphate*	Cobalamin Metabolism	RXN0653	RSP0430
Heme*	Heme Metabolism	RXN0632	RSP1197
Ethanolamine*	Glycerophospholipid Metabolism	RXN0378	RSP0113

*Competitors for sub-optimal growth conditions only.

** Secretion of central metabolism intermediates pyruvate, fumarate, and malate were blocked, assuming that cells are programmed to reuse these compounds as carbon sources even after secretion. This was based on observations with batch cultures [3], but might not hold true for continuous cultures.

FBA has also enabled us to model the flow of electrons through the aerobic respiratory chain. *R. sphaeroides *possesses two cytochrome oxidases (Cox): aa3-type cytochrome c oxidase and cbb3-type cytochrome c oxidase, which carry out the same reaction but have different oxygen affinities. *R. sphaeroides *also possesses two quinol oxidases (Qox) - QoxBA and QxtAB - that provide a less energetically efficient means for recycling reduced electron carriers [56]. While it is possible that both Cox and Qox could be used simultaneously, maximization for biomass during FBA simulations results in only the more efficient Fbc-Cox portion of ETC being utilized. Mutational analysis has shown that deletion of either Qox or Qxt has no effect on aerobic growth rate [56], which is in agreement with the predictions of iRsp1095. In addition, mutation of the Fbc complex, which is predicted to redirect flux through the Qox pathway, results in a two fold increase in doubling time experimentally, which is almost identical to the predictions of iRsp1095 (see results and [56]). Furthermore, the loss of both Cox and Qox activity is also correctly predicted by iRsp1095 to be lethal under aerobic conditions. Thus, iRsp1095 accurately models the flux distribution through the aerobic respiratory chain. The reversibility of the NADH dehydrogenase reaction predicted by iRsp1095 and its essentiality during photosynthetic growth has previously been observed in the closely related photosynthetic bacterium *Rhodobacter capsulatus *[55,57]. Furthermore, conclusions on the essential role of NADH dehydrogenase in synthesizing NADH for anabolic processes under photosynthetic conditions are in agreement with predictions of iRsp1095. Unlike *R. capsulatus, R. sphaeriodes *is predicted to contain two isozymes of the NADH dehydrogenase complex, with genes encoding both enzymes being expressed during photoheterotrophic growth [58]. Experimental analysis of the role of each NADH dehydrogenase isozymes during anaerobic growth in *R. sphaeroides *is required to compare with the predictions of iRsp1095.

Finally our simulations predict that several alternative optimal solutions are often possible under any given condition, reinforcing the need to analyze the space of alternate optima [21-23]. The diverse metabolic capabilities of *R. sphaeroides *reinforces the challenge of making accurate predictions about condition-dependent metabolic fluxes as not all feasible functional states are relevant to wild type cells. Thus, to obtain improved predictions of the flux distributions through the network of wild-type *R. sphaeroides*, additional constraints on iRsp1095 will be required.

## Conclusions

iRsp1095 represents the first comprehensive genome-scale metabolic reconstruction for a facultative photosynthetic bacterium. This genome-scale reconstruction has enabled us to examine the metabolic capabilities of this purple non-sulfur bacterium. Our modeling results predict that *R. sphaeroides *possesses multiple pathways that could be exploited as electron sinks during photoheterotrophic growth, though experimental results suggest many of these are silent in wild type cells. Other results predict that additional gains in H_2 _production are possible as the production capacity of wild type cells is only about two-thirds of the theoretical maximum, with pathways and reactions that could increase production predicted using iRsp1095. An alternative route for CO_2 _fixation, the ethylmalonyl pathway, was predicted using iRsp1095. This prediction could potentially resolve the question of how *R. sphaeroides *assimilates CO_2 _in the absence of RubisCO. iRsp1095 also predicts the reversibility of the NADH dehydrogenase complex and its essentiality during photoheterotrophic growth on succinate, where it plays a key role in oxidation of ubiquinol. Further experimental work is needed to confirm these predictions and improve our understanding of the metabolic network of this and possibly other related bacteria. Finally, quantitative predictions made using iRsp1095 showed good agreement with experimental observations, verifying the utility of the model and highlighting the potential for its use in quantitative analysis of *R. sphaeroides *metabolism.

## Methods

### Constraint-based simulations

A stoichiometric matrix, S_m × n_, was generated from the reconstruction with the rows (m) representing the metabolites, the columns (n) representing the reactions and the entries in the matrix representing the stoichiometric coefficients for metabolites involved in each reaction. Flux balance analysis (FBA) [19] was used to simulate *in silico *growth by solving the linear programming problem:(1)(2)(3)

where v_Biomass _is the flux through biomass objective function (BOF); **v **is the vector of steady state reaction fluxes; and **v**_min _and **v**_max _are the minimum and maximum allowable fluxes. The values in **v**_min _and **v**_max _were set to -1000 and 1000 mmol/g DW h for reversible reactions, 0 and 1000 mmol/g DW h for forward only reactions, and -1000 and 0 mmol/g DW h for backward only reactions, respectively. During simulation all exchange reactions were assigned as being forward only (allowing metabolites to be secreted into the medium but no taken up), except the exchange reactions for media components required by the cell for growth, which were set to measured values for limiting substrates - carbon and nitrogen sources, or allowed to be freely exchanged with the extracellular space, i.e., -1000 ≤ v ≤ 1000. In addition, the non-growth associated ATP maintenance limit was set to 8.39 mmol/gDW h [38].

Flux variability analysis (FVA) was carried out as described in [21] by first determining the flux through the BOF using FBA, then determining the maximum and minimum possible fluxes through each of the reactions in the network, while the BOF is fixed at the FBA optimum, using equations (4) and (5) below.(4)(5)

where Z is the optimal flux through the BOF predetermined using FBA.

Alternate optima analysis was conducted as described in [23], using a mixed integer linear programming algorithm that is a modification of that previously used in [22], which prevents revisiting of already identified optimal solutions. In addition to the FBA constraints outlined above (i.e., Equations 1, 2 and 3), the alternate optima algorithm requires the implementation of the following additional constraints:(6)(7)(8)(9)

where y_i_, w_i _**∈** {0, 1}, NZ is a set of indices that keeps track of non-zero fluxes of interest for each iteration j. During each iteration through j, at least one of these fluxes of interest v_i _with a non-zero value must be set to zero and thus y_i _for the corresponding flux is set to 1 (Equation 6). When y_i _takes the value 1, w_i _is forced to 0 (Equation 8), forcing the upper and lower bounds of v_i _to zero (Equation 9). Equation 7 ensures that previously identified optima are not revisited by forcing at least one non-zero flux to have a zero value for the next iteration. Fluxes of interest used in our alternate optima analysis were restricted to those observed to be involved in redox balancing or for which we had experimental measurements for comparison (RXN1205, RXN0222, RXN0109, RXN1427, RXN1308, RXN1425, RXN1441, RXN1121, and RXN0681 - see Additional File 2 - Table S1 contains reaction details). The use of this set of reactions proved more efficient at sampling the optimal solution space for desired solutions, than using all the reactions in iRsp1095, as it identified an equivalent number or more optimal solutions in which all 3 measured metabolites (i.e., CO_2_, H_2 _and PHB) had non-zero fluxes.

Deletion analysis was initially carried out at the reaction level by sequentially setting the flux of each reaction to zero, then using FBA to compute the optimal growth rate. Reactions which led to the production of no biomass were considered essential. At the gene level, the fluxes of all reactions associated with a particular gene were set to zero and FBA used to compute the optimal growth rate. Genes encoding proteins whose reactions were required for the formation of biomass, and for which there existed no isozymes in the model, were considered essential.

For analysis of potential carbon, nitrogen, phosphorus and sulfur sources utilized in iRsp1095, simulations were conducted using SIS as the baseline media, which contains succinate, ammonium, phosphate, and sulfate as the only the sources of carbon, nitrogen, phosphorus, and sulfur, respectively. To test a different source, the original metabolite was removed and replaced with the metabolite to be tested. When needed, temporary sink reactions [20] were added for each metabolite to be tested and these reactions were removed at the completion of the analysis. Metabolites which resulted in the predicted growth rate greater than 0 were considered as potential growth substrates. All simulations were conducted under the GAMS programming environment (GAMS Development Corporation, Cologne, Germany) using the CPLEX solver.

### Continuous Cultures

To obtain steady state growth data for FBA, wild type *R. sphaeroides *2.4.1 was cultured in 20 mL chemostats at ~30°C, either continuously illuminated by an incandescent light source for photosynthetic growth (~10 W/m^2^, as measured with a Yellow-Springs-Kettering model 6·5-A radiometer through a Corning 7-69 filter), or continuously aerated (4 mL/min from a compressed air cylinder) in dark conditions for aerobic growth. The turbidity of photosynthetic cells was monitored using a Klett-Summerson photoelectric colorimeter (Klett MFG Co., NY), while that of aerobic cultures was measured spectrophotometrically at 600 nm wavelength with a UV-1601 Spectrophotometer (Shimadzu Scientific, Columbia, MD). Reactors were started in batch mode [3] until cells reached >100 Klett units or >300 O.D. at 600 nm, and were then continuously fed with medium using Masterflex peristaltic pumps (Cole-Palmer Instrument Co., Vernon Hills, IL). To reach the desired retention time, an appropriate amount of medium was replaced by 5-min continuous pumping every hour. Reactors were checked approximately every 12 hours, and when necessary, pumping was manually adjusted to correct small changes in reactor volume due to marginal imbalances of inflow and outflow. Cultures were grown for at least 5 retention times and stopped when steady state was established as evidenced from constant turbidity measures or -in case of some photosynthetic cultures- constant gas rates. All reactors were fed with Sistrom's minimal medium [41] containing one of the following pairs of carbon and nitrogen sources in the respective order: 33.9 mM succinate and 7.5 mM ammonia, 33.9 mM succinate and 8.1 mM glutamate, 19.8 mM glucose and 8.1 mM glutamate, and 26.6 mM glutamate as both carbon and nitrogen sources.

### Biomass composition analysis

Cultures were centrifuged (6,000 rpm, 12 mins, 4°C) to obtain cell pellets for biomass analysis. Cell pellets pooled from several chemostats were resuspended in 1X SIS medium, mixed, and distributed into different subsamples for measuring individual biomass components. The major cellular components measured were protein, DNA, RNA, cell wall, lipids, bacteriochlorophyll, carotenoids, glycogen and PHB.

Total cellular protein was quantified via the Lowry assay [59,60], while total DNA and RNA were determined spectrophotometerically after phenol/chloroform and perchloric acid extraction respectively [61]. Total cellular lipid content was estimated using the sulfo-phospho-vanillin assay on crude lysates [62], while the phospholipid component was determined by total phosphorus assay on lipids extracted via standard chloroform/methanol extraction [63]. Total cellular bacteriochlorophyll was determined spectrophotometrically at 770 nm following acetone/methanol extraction. Bacteriochlorophyll levels were used in estimating cellular carotenoid content based on the previously determined 2:1 ratio of bacteriochlorophyll to carotenoids in the B800-850 complex of *R. sphaeroides *[64].

The PHB content of cells was determined by GC-MS (GC-2010 gas chromatograph coupled to a QP-2010S mass spectrometer detector; Shimadzu Scientific) [3,65]. Cellular glycogen content was determined by digestion of glycogen in cellular extracts to glucose using amyloglucosidase (Sigma-Aldrich) and quantification of glucose, using a glucose (HK) assay kit (Sigma-Aldrich). Identically treated dilutions of glycogen (Sigma-Aldrich) were used as standard for quantification. Cell wall composition of biomass was assumed to be similar to that of *E. coli *[38]. Finally, the fraction of inorganic material was based on ash content in previous biomass analyses of closely related species [66].

### Biomass reaction and net cell dry weight (dW)

The biomass reaction of the metabolic model was formulated using major biomass components as detailed in Additional File 3. Since PHB and glycogen varied significantly based on carbon source and growth conditions, they were not included in the biomass reaction. Instead, they were modeled via the addition of demand reactions to allow their accumulation during simulation. Accordingly, the normalization of all fluxes was done using a dry weight (dW) calculation that excludes PHB and glycogen from the cell mass estimate. For this, we calculated dW using a chemical oxygen demand (COD) mass balance approach [3] as shown in Equation 10, where COD_biomass _represents the overall measurement of COD in cells, COD_PHB _and COD_glycogen _represent COD of PHB and glycogen obtained from experimental measurements and theoretical COD/weight ratios (1.67 mgCOD/mgPHB and 1.18 mgCOD/mg glycogen), and θ is the COD/weight ratio for the cell mass according to the biomass reactions established in this study (θ is 1.62 for photosynthetic and 1.56 for aerobic cultures).(10)

### Other Analytical Measurements

Substrate uptake rates of all cultures and gas composition of the headspace in phototrophic chemostats were measured using previously described protocols [3] (see Additional File 1). No gas evolution measurements were taken for aerobic cultures as they were continuously aerated. However, the amount of O_2 _uptake, an important parameter in the FBA of aerobic growth, was indirectly measured by COD, which was previously used for analysis of electron fate in photosynthetic cultures of *R. sphaeroides *[3]. Briefly, the COD of the medium (inflow) and of the reactor effluent were measured using HACH High Range (0-1500 mg/L) COD kits (HACH Company, Loveland, CO), and the difference between medium and effluent (including cells) gave the estimated O_2 _utilization by the cells due to the mass balance of electrons [3].

## Competing interests

The authors declare that they have no competing interests.

## Authors' contributions

SI and LSY participated in the reconstruction, curation and assessment of iRsp1095. US, ASG and LSY set up *R. sphaeroides *continuous cultures and obtained analytical data. SI and LSY participated in the determination of the *R. sphaeroides *biomass composition. JLR provided code samples for metabolic modeling. TJD and DRN conceived of project and coordinated research. SI wrote paper with critical reading and revisions by LSY, JRL, DRN and TJD. All authors read and approved the final manuscript.

## Supplementary Material

Additional file 1 **Supplemental materials and methods **Additional File 1 contains details of the model reconstruction and refinement process, as well as details of GC/MS and supernatant analysis. Click here for file

Additional file 2 **iRsp1095 Metabolic Reconstruction **Additional File 2 contains 12 tables. Table S1 list all the reactions in iRsp1095 including official reaction names, reaction stoichiometry, GPR assignments, directionality assignments and localization information. Table S2 contains information about metabolites in the model. Table S3 contains thermodynamic calculations used in the determined reaction directionality. Table S4 lists reactions constrained to eliminate SBCs. Table S5 lists the genes present in the model. Table S6 summarizes simulated growth phenotypes with various carbon sources. Table S7 lists all the references used in curation of iRsp1095. Table S8 lists reactions added to the model based on metaSHARK analysis. Table S9 details the compositions of SIS minimal media. Table S10 lists essential genes identified by FBA analysis. Table S11 list the gap filling reactions added to iRsp1095. Table S12 contains as description of the confidence scores assigned to the reactions in iRsp1095. Click here for file

Additional file 3 **Biomass calculations **This contains calculations of the coefficients of the biomass precursors based on determined contribution to biomass and genomic information for aerobic and photosynthetic growth. Click here for file

Additional file 4 **iRsp1095 in SBML **SBML format of iRsp1095 for distribution and use in other modeling environments. Click here for file

Additional file 5 **Sensitivity analysis **Additional File 5 contains additional sensitivity analysis conducted to assess the effects of light, biomass composition and P/O ratio on growth and metabolite production rates. Click here for file
