# Supplementary material for: iRsp1095: A genome-scale reconstruction of the Rhodobacter sphaeroides metabolic network
Source: BMC Syst Biol. 2011 Jul 21;5:116. doi: 10.1186/1752-0509-5-116 (PMC3152904; doi:10.1186/1752-0509-5-116)
Supplement: Additional file 1 — Supplemental materials and methods Additional File 1 contains details of the model reconstruction and refinement process, as well as details of GC/MS and supernatant analysis. [file 1752-0509-5-116-S1.DOC]

**Supplemental Material and Methods**

***Network Reconstruction***

We began the construction of iRsp1095 by extracting all the archived *R. sphaeroides 2.4.1* metabolic reactions from KEGG [1] and generating a draft reconstruction. In addition, an alternative draft reconstruction was generated using metaSHARK [2] and compared with the KEGG draft reconstruction via enzyme commission (EC) numbers. Additional enzymatic reactions identified by metaSHARK were added to the reconstruction when they did not lead to additional dead end metabolites (Additional File 2 – Table S8). Each reaction in the reconstruction was then manually curated against literature for experimental evidence of their existence in *R. sphaeroides* (Additional File 2 - Table S7), with each reaction given a confidence score between 0 and 4, as outlined in [3](Additional File 2 - Table S1, Table S12), based on quality of supporting evidence for its presence in the reconstruction. Non-metabolic reactions, dead end side reactions and duplicate reactions were removed from the reconstruction and the remaining reactions computationally balanced for mass and charge. Gene-Protein-Reaction (GPR) assignments were systematically carried out first by using literature references when these were available, then via the use of Pfam functional domain [4] and sequence homology (BLASTp) searches, using E-value cutoffs of 10^-5^ [5]. Homologous proteins with equivalent functional domains attached to particular reaction in the draft reconstruction, were tentatively assigned as isozymes, while proteins with distinct functional domains or non-homologous proteins attached to a particular reaction were tentatively assigned as subunits of a multi-protein complex. The reconstruction accounts for 3 compartments: the extracellular space, the cytoplasm and the periplasm. Cellular localization of the various reactions were assigned based on Psortb v3.0.2 [6] analysis of their corresponding proteins, with unknown predictions being re-assigned as cytoplasmic. The localization predictions were further updated based on experimental evidence where necessary. Metabolites were allowed to be shuttled between compartments via known or inferred transporters.

***Reaction Directionality***

The reversibility of the reactions in the network were determined via a combination of thermodynamic and heuristic calculations/assumptions, which have previously been used by Henry et al [7]. These calculations depend on estimation of Gibbs free energies of formation (Δ_f_G'°) of the metabolites, and then using these values in the calculation of the Gibbs free energy of reaction (Δ_r_G'°). Δ_r_G'° is then utilized in calculating the Gibbs free energy of reactions at 1mM concentration of metabolites (Δ_r_G'^m^), as well as minimum (Δ_r_G'_min_) and maximum (Δ_r_G'_max_) Gibbs free energy of reactions at physiologically relevant extremes of product and reactant concentrations [7-10]. The Δ_f_G'° of 619 (78%) of the metabolites in the reconstruction were determined using the WebGCM online tool [10] (Additional File 2 - Table S2) and these values used in the calculation of the Δ_r_G'°, Δ_r_G'^m^, Δ_r_G'_min_ and Δ_r_G'_max_ for 897 (78%) of the reactions in the network. Heuristic rules which were used to augment thermodynamic calculations for the assignment reaction directionality were based on known irreversible biochemical reactions which include ABC transport, carboxylation, phosphorylation, CoA/ACP ligase and ATP-dependent reactions [7]. The reversibility score, S_rev_, was calculated as previously described in [7] using CO_2_, HCO_3_-, CoA, ACP, phosphate, and pyrophosphate as low energy substrates and their directions determined based on reversibility rules developed in [7] (Additional File 2 - Table S3). An additional 46 reactions were assigned as irreversible based only on heuristic assumptions [7]; these correspond to ABC transporters, tRNA charging and known spontaneous reactions.

The initial model was analyzed for SBCs [3, 11], which are internal network loops that carry flux in a closed system with no net production or consumption of metabolites. SBCs generally result from insufficient constraints on the metabolic network and can lead to erroneous flux distributions from linear constraint-based analysis like FBA [3, 12]. Furthermore, *R. sphaeroides* is known or predicted to possess shared pathways or dual enzymes (oxygen-dependent or independent) for reactions in the biosynthesis of key metabolites [13] (e.g., protoporphyrinogen IX – an intermediate in heme biosynthesis, divinylprotochlorophyllide – an intermediate in bacteriochlorophyll biosynthesis, and cobalamin), which if left unconstrained, can lead to erroneous generation of oxygen (O_2_) by the model during simulation of anaerobic growth. SBCs were manually removed from iRsp1095 by identifying the minimal number of additional directionality constraints required to prevent cyclic flux via these loops, which could be corroborated either by thermodynamic analysis or by reaction databases such as KEGG and published manually curated metabolic reconstructions.

***Network connectivity***

In order for the reconstructed model to predict the production of biomass, gaps in the metabolic network needed to be filled with the appropriate missing reactions. Automated gapfilling approaches, such as gapfill/gafind [14] are available, which enable filling in of network gaps based on comparison with reference databases. However, we sought to incorporate only the minimal number of gapfilling reactions into the *R. sphaeroides* network, to limit the number of uncharacteristic behaviors exhibited by the network. Thus, we carried out gap filling first by computationally analyzing the topology of the network to identify dead end metabolites, which did not constitute end products of biosynthetic pathways, and then one of the following steps was taken:

1. add a gap filling reaction that reconnects it to the rest of the network
2. add a transport reaction to allow the uptake or secretion of the metabolite into the extracellular space
3. remove the reaction from the network if both reactants and products are dead end metabolites (these reactions were generally as a result of side reactions catalyzed by enzymes - which catalyze other reactions in the network - and would require multiple reactions to be restored to the network)

This approach is very similar to [14], but involved more manual input as greater care was taken with the reactions filled in. In addition to this, *R. sphaeroides,* based on minimal growth requirements, was assumed to be capable of de novo synthesis of certain cofactors (such as tetrahydrofolate). However, in some cases, the required reactions for these pathways were missing from the initial reconstruction as the genes that produce this functionality are still unknown. These reactions were filled in with the appropriate reactions via the analysis of KEGG network maps.

***Supernatant Analysis***

Culture supernatants were filtered and analyzed to obtain substrate uptake values using previously described protocols [15]. All organic acids were measured using GC-MS (see above) and glucose was quantified with HPLC (model 10A-vP equipped with an RID-10A refractive index detector, Shimadzu Scientific).

***Gas Analysis***

To identify and quantify the gas produced by photosynthetic cultures, protocols described in [15] were followed. In brief, reactors were connected to an AER-200 respirometer (Challenge Technology, Springdale, AR), and the composition of the gas in the reactor head space was determined using a GC-2014 gas chromatograph (Shimadzu). The headspace of gas producing cultures was dominated by H_2_ and CO_2_. The concentration of aqueous H_2_ and CO_2_ in photosynthetic cultures were calculated based on Henry’s law using k_H_ values of 1282 L.atm/mol and 29.4 L.atm/mol, respectively [16]. While the total H_2_ production rate was obtained as the sum of H_2_ flow rate in gas (headspace) and aqueous (effluent) forms, the same addition does not provide the total CO_2_ production, as a significant portion of the CO_2_ is chemically converted to HCO_3_^-^ under the pH conditions of the reactors (pH=6.7-7.8). Therefore, using a pK_a_ value of 6.352, the concentration of HCO_3_^-^ was calculated from equilibrium chemistry [17]. Consequently, the total CO_2_ production rate was obtained as the sum of CO_2_ flow rate in gas (headspace), aqueous (effluent), and HCO_3_^-^ (effluent) forms.

**References**

1. Kanehisa M, Goto S, Kawashima S, Nakaya A: **The KEGG databases at GenomeNet**. *Nucleic Acids Res* 2002, **30**(1):42-46.

2. Pinney JW, Shirley MW, McConkey GA, Westhead DR: **metaSHARK: software for automated metabolic network prediction from DNA sequence and its application to the genomes of *Plasmodium falciparum* and *Eimeria tenella***. *Nucleic Acids Res* 2005, **33**(4):1399-1409.

3. Thiele I, Palsson BO: **A protocol for generating a high-quality genome-scale metabolic reconstruction**. *Nat Protoc* 2010, **5**(1):93-121.

4. Bateman A, Coin L, Durbin R, Finn RD, Hollich V, Griffiths-Jones S, Khanna A, Marshall M, Moxon S, Sonnhammer EL *et al*: **The Pfam protein families database**. *Nucleic Acids Res* 2004, **32**(Database issue):D138-141.

5. Suthers PF, Dasika MS, Kumar VS, Denisov G, Glass JI, Maranas CD: **A genome-scale metabolic reconstruction of *Mycoplasma genitalium*, iPS189**. *PLoS Comput Biol* 2009, **5**(2):e1000285.

6. Yu NY, Wagner JR, Laird MR, Melli G, Rey S, Lo R, Dao P, Sahinalp SC, Ester M, Foster LJ *et al*: **PSORTb 3.0: improved protein subcellular localization prediction with refined localization subcategories and predictive capabilities for all prokaryotes**. *Bioinformatics* 2010, **26**(13):1608-1615.

7. Henry CS, Zinner JF, Cohoon MP, Stevens RL: **iBsu1103: a new genome-scale metabolic model of *Bacillus subtilis* based on SEED annotations**. *Genome Biol* 2009, **10**(6):R69.

8. Feist AM, Henry CS, Reed JL, Krummenacker M, Joyce AR, Karp PD, Broadbelt LJ, Hatzimanikatis V, Palsson BO: **A genome-scale metabolic reconstruction for *Escherichia coli* K-12 MG1655 that accounts for 1260 ORFs and thermodynamic information**. *Mol Syst Biol* 2007, **3**:121.

9. Henry CS, Jankowski MD, Broadbelt LJ, Hatzimanikatis V: **Genome-scale thermodynamic analysis of *Escherichia coli* metabolism**. *Biophys J* 2006, **90**(4):1453-1461.

10. Jankowski MD, Henry CS, Broadbelt LJ, Hatzimanikatis V: **Group contribution method for thermodynamic analysis of complex metabolic networks**. *Biophys J* 2008, **95**(3):1487-1499.

11. Price ND, Famili I, Beard DA, Palsson BO: **Extreme pathways and Kirchhoff's second law**. *Biophys J* 2002, **83**(5):2879-2882.

12. Price ND, Thiele I, Palsson BO: **Candidate states of *Helicobacter pylori*'s genome-scale metabolic network upon application of "loop law" thermodynamic constraints**. *Biophys J* 2006, **90**(11):3919-3928.

13. Hunter CN, Daldal, F., Thurnauer, M. C. and Beatty, J. T.: **The Purple Phototrophic Bacteria**, vol. 28: Springer; 2009.

14. Satish KV, Dasika MS, Maranas CD: **Optimization based automated curation of metabolic reconstructions**. *BMC Bioinformatics* 2007, **8**:212.

15. Yilmaz LS, Kontur WS, Sanders AP, Sohmen U, Donohue TJ, Noguera DR: **Electron partitioning during light- and nutrient-powered hydrogen production by *Rhodobacter sphaeroides***. *Bioenerg Res* 2010, **Volume**(1):55 - 66.

16. Sander R: **Compilation of Henry's Law Constants for Inorganic and Organic Species of Potential Importance in Environmental Chemistry (Version 3)**. In*.*; 1999.

17. Benjamin MM: **Water Chemistry**, 1st Edition edn: McGraw-Hill Companies; 2002.
