# Supplementary material for: iRsp1095: A genome-scale reconstruction of the Rhodobacter sphaeroides metabolic network
Source: BMC Syst Biol. 2011 Jul 21;5:116. doi: 10.1186/1752-0509-5-116 (PMC3152904; doi:10.1186/1752-0509-5-116)
Supplement: Additional file 5 — Sensitivity analysis Additional File 5 contains additional sensitivity analysis conducted to assess the effects of light, biomass composition and P/O ratio on growth and metabolite production rates. [file 1752-0509-5-116-S5.DOC]

**Sensitivity analysis**

***Biomass composition***

To determine the sensitivity of our FBA predictions to the composition of the biomass objective function (BOF), we conducted sensitivity analyses by varying the percent contributions of the individual macromolecular components of the BOF. The resultant collection of BOFs were generated by varying the percent composition of protein (40 – 70%), lipids (15 – 30%), RNA (3 – 12%) and pigments (1 – 5%), while still ensuring the percent contributions by mass summed up to 100%. FBA was then used to determine the maximal growth rate predicted using each of the BOFs, for succinate uptake rates (SUR) between 1 and 10 mmol/g DW h (Fig S1-A) during photoheterotrophic growth. For analysis of the production rate of metabolic products, nitrogen limiting conditions were required, so the ammonia uptake rate was set to 1 mmol/g DW h, which is within the range experimentally observed uptake rates for this metabolite based on chemostat data (see Fig 3a in results section).

**Figure S1. Biomass composition sensitivity analysis.** The graphs illustrate the results of sensitivity analysis conducted to determine the effect of biomass composition of the BOF on growth rate (GR) (Fig S1-A) and the production rates of H_2_ (Fig S1-B), PHB (Fig S1-C) or CO_2_ (Fig S1-D). Min. prod. rate and min. GR refers to the minimum observed maximum production (or growth) rates across the collection of BOFs, while max. prod. rate and max. GR refer the maximum observed maximal production (or growth) rates across the collection of BOFs. The dotted lines represent the region of SUR that correspond to that observed in chemostat cultures maintained under photoheterotrophic conditions.

The results show that varying the macromolecular contributions of the BOF has very little impact (7.5% maximum difference) on the predicted maximal growth rate as previously observed with an *E. coli* model [1]. On the other hand, the maximal production rates of H_2_, PHB and CO_2_ (at maximum growth rate) do show significant differences between the various BOFs (up to a 2.3 fold difference across the range of BOFs), with the most significant differences observed at SUR between 1.5 and 4 mmol/g DW h (dotted lines), which represent the range of experimentally observed SUR in chemostats harboring photoheterotrophic *R. sphaeroides* 2.4.1 cultures (Fig S1-B, S1-C, S1-D). However, it must be noted that the actual magnitude of these differences (2 mmol/g DW h or less) are not particularly large, thus it needs to be determined detrimental they will be to the value of the prediction. Nevertheless, these analyses highlight the importance of having a relatively accurate BOF in order to correctly predict of important cellular metabolic outputs besides growth rate, when carrying out optimization using biomass production as the objective function.

***Light uptake***

As all our simulations of photoheterotrophic growth were conducted in carbon/nitrogen limiting conditions with light uptake left unconstrained, we carried out robustness analysis to assess the effect of photo uptake on the maximum production rate of H_2_, PHB and CO_2_ at optimal growth. The photon uptake rate was varied between 15 and 90 mmol/g DW h, while SUR and ammonia uptake rates were set to experimentally determined values measured during photoheterotrophic growth (Fig S2).

**Figure S2. Light sensitivity analysis**. The effect of light uptake rate on the growth rate and the production rates of H_2_, PHB and CO_2_

As might be expected, the optimal growth rate increases as photon uptake increases (upper panel). Fig S2 shows there is an inverse correlation between the growth rate and CO_2_ production as the light uptake rate increases, with maximum growth achieved at a photon uptake rate of about 45 mmol/g DW h. Only after the optimal growth rate is achieved, given the supplied nutrients, does H_2_ and PHB production begin, as excess reducing power can now be utilized in the synthesis of these metabolites. The ability to simulate the production of H_2_ and PHB is dependent on the fact that optimal growth rate is limited by nitrogen (in this case ammonia), thus allowing the excess succinate supplied to the model to be utilized in the production of these metabolites. The data illustrate that sufficient light is required for both optimal growth and metabolite production but that there is an upper limit beyond which additional photon uptake no longer increases metabolite production due to constraints on nutrient uptake.

**P/O ratio**

Sensitivity analysis was also carried out to determine the optimal P/O ratio (i.e. the rate of ATP synthesis to oxygen consumption) predicted by model during simulation of aerobic growth, as well as to determine the effect on growth rate of varying the P/O ratio. P/O ratios ranging from 1.2 to 2.5 were set as constraints for FBA analysis, while the SUR was varied between 1 and 10 mmol/g DW h. Depending on the SUR, the difference in growth rate resulting from this range of P/O ratios varied from 31 to 78% (35 – 40% within the range of experimentally observed SUR) (Fig S3), indicating the P/O ratio can have a significant impact on growth rate, a result previously observed with current *E. coli* model [1].

**Figure S3. P/O ratio sensitivity analysis**. The plot illustrates the effect on growth rate of varying the P/O ratio during aerobic growth. Min. prod. rate and Min. GR refers to the minimum observed maximum production (or growth) rates across the range of P/O ratios tested respectively, while max. prod. rate and max. GR refer the maximum observed maximal production (or growth) rates across the range of P/O ratios. The dotted lines represent the region of SUR that correspond that generally observed from cells in continuous culture.

**References**

1. Feist AM, Henry CS, Reed JL, Krummenacker M, Joyce AR, Karp PD, Broadbelt LJ, Hatzimanikatis V, Palsson BO**: A genome-scale metabolic reconstruction fo*r Escherichia co*li K-12 MG1655 that accounts for 1260 ORFs and thermodynamic informati**on*. Mol Syst Bio*l 2007**,** 3:121.
